# DIA-PRM Proteomic Analysis of Phlegm-Dampness Constitution with Glucolipid Metabolic Disorders by the Intervention of Hua Tan Qu Shi Recipe

**DOI:** 10.1155/2022/6464431

**Published:** 2022-12-23

**Authors:** Yuanyuan Li, Jiayi Ma, Shuxian Sun, Lingru Li, Huirong Song, Jing Xia, Houqin Li, Dandan Hu, Cheng Ni

**Affiliations:** ^1^Center for Studies in Constitution Research of Traditional Chinese Medicine, Beijing University of Chinese Medicine, Beijing 100029, China; ^2^Institute of Basic Theory for Chinese Medicine, China Academy of Chinese Medical Sciences, Beijing 100700, China; ^3^The Gulou Hospital of Traditional Chinese Medicine of Beijing, Beijing 100009, China; ^4^National Institute of TCM Constitution and Preventive Medicine, Beijing University of Chinese Medicine, Beijing 100029, China

## Abstract

**Background:**

Phlegm-dampness constitution as one of nine constitutions in traditional Chinese medicine (TCM) has been a high risk factor for glucolipid metabolic disorders (GLMD). Based on our previous findings, Hua Tan Qu Shi recipe (HTQSR) could effectively improve metabolic indicators of GLMD by targeting on phlegm-dampness constitution. However, the proteomic mechanisms of GLMD with the treatment of HTQSR targeting on phlegm-dampness constitution remain unknown.

**Methods:**

Clinical participants from phlegm-dampness constitution with the prediabetic state (T), phlegm-dampness constitution with marginally elevated blood lipids (Z), and phlegm-dampness constitution before sickness (W) were included in this study, who orally took HTQSR for 12 weeks and, respectively, marked AT, AZ, and AW. Data-independent acquisition (DIA) and parallel reaction monitoring (PRM) were performed to identify the differential proteins; then, Venn analysis was used to investigate coexpressed and coregulated proteins. In addition, ingenuity pathway analysis (IPA) software was utilized to explore the related pathways and diseases and biofunctions.

**Results:**

LXR/RXR activation, acute phase response signaling, and production of nitric oxide and reactive oxygen species in macrophages were obviously activated between the T and AT groups, as well as the Z and AZ groups. In contrast, these three pathways were inhibited between the W and AW groups. Importantly, one coexpressed and coregulated differential protein, B2MG, was validated by PRM among all groups.

**Conclusions:**

This work firstly reported the underlying proteomic mechanisms of GLMD with the treatment of HTQSR targeting on phlegm-dampness constitution, indicating that intervention of phlegm-dampness constitution might be a novel strategy for the preventive treatment of GLMD.

## 1. Introduction

The stability of glucose and lipid metabolism is essential for maintaining the function of various organs in the body [1]. With the improvement of living standards, the incidence of glucolipid metabolic disorders (GLMD), such as hyperlipidemia and diabetes, is increasing in the world [2]. Moreover, GLMD could damage different body organs, especially the kidneys, heart, eyes, and nerves [3]. At present, the single-target treatment strategy applied in clinical practice against GLMD exhibits the poor control of overall blood lipid, blood glucose, blood pressure, and other indicators [4]. Therefore, it is very necessary to develop a novel strategy to effectively treat GLMD.

Phlegm-dampness constitution as one of nine constitutions in TCM is a group of individuals with the dysfunctional and subhealthy status [5], who have the common characteristics including slippery pulse, abundant sputum, chest distress, obesity, oily skin in the face, sticky and sweet taste in the mouth, and greasy and soft lower abdomen [6]. Phlegm-dampness constitution is one high-risk factor for chronic metabolic disorders [7]. Multinomial logistic regression analysis of 3748 participants has indicated that phlegm-dampness was positively correlated with overweight and obesity [8]. Epidemiologic studies have reported that phlegm-dampness constitution is highly related to diabetes and metabolic syndromes (MS) [9]. Genomic studies have illustrated that phlegm-dampness constitution has the molecular basis of metabolic disorder and is the potential risk for arteriosclerosis and thrombosis; the individuals are susceptible to hyperlipemia and diabetes [10], and various genes in the phlegm-dampness constitution are involved in glucose and lipid metabolism pathways [11]. In addition, single-nucleotide polymorphisms and methylation investigation have also confirmed that phlegm-dampness constitution is closely related to GLMD [12]. According to these findings, intervention of phlegm-dampness constitution by the TCM might provide a potential treatment for glucolipid metabolic disorders.

**Figure 1:**
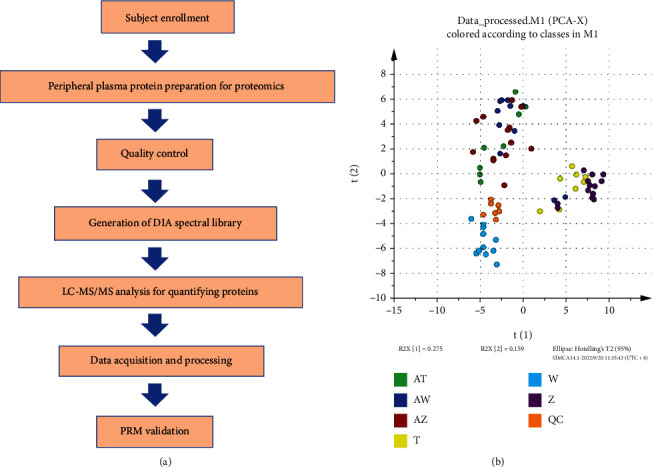
Overview of proteomic profiling. (a) Proteomic profiling procedure; (b) score plot of the unsupervised PCA model for all samples among the groups. W: phlegm-dampness constitution before sickness without the treatment; AW: phlegm-dampness constitution before sickness with the treatment of HTQSR for 12 weeks; T: phlegm-dampness constitution with the prediabetic state without the treatment; AT: phlegm-dampness constitution with the prediabetic state with the treatment of HTQSR for 12 weeks; Z: phlegm-dampness constitution with marginally elevated blood lipids without the treatment; AZ: phlegm-dampness constitution with marginally elevated blood lipids with the treatment of HTQSR for 12 weeks; QC: the mixture of all the samples.

Hua Tan Qu Shi recipe (HTQSR), also called phlegm-dampness constitution conditioning formula, is a clinical prescription invented by Professor Wang Qi. The patent number of HTQSR is ZL201410538335.9. In 2019, our group preliminarily confirmed that HTQSR could decrease the scores of phlegm-dampness constitution, ameliorate symptoms of phlegm-dampness retention, and improve metabolic indicators of GLMD [13]. However, no references reported the proteomic mechanisms of GLMD with the treatment of HTQSR targeting on phlegm-dampness constitution. In this work, HTQSR is clinically used to treat the glucolipid metabolic disorders via regulating the phlegm-dampness constitution. The possible mechanisms against glucolipid metabolic disorders caused by phlegm-dampness constitution are investigated by the DIA-PRM proteomic analysis. Overview of proteomic profiling was displayed in Figure 1.

## 2. Materials and Methods

### 2.1. Ethical Approval

The Ethics Committee of the Beijing University of Chinese Medicine (Beijing, China) approved all clinical experiments, and the approval number was 2019BZYLL0310. Written informed consent was obtained from all subjects and all these experiments were conducted in accordance with approved guidelines.

### 2.2. Subject Enrollment

Participants in this work aged from 18 to 50 years were recruited from communities in Beijing. Subjects with phlegm-dampness constitution were diagnosed based on the “Traditional Chinese Medicine Constitution Classification and Distinguishing Scale (2009 Edition).” Subjects with marginally elevated blood lipids were diagnosed based on the “Guidelines for the Prevention and Treatment of Dyslipidemia in Adults in China (2016 Revised Edition).” Subjects with prediabetes were diagnosed based on the “Guidelines for the Prevention and Control of Type 2 Diabetes in China (2017 Edition).” The exclusion criteria for participants in this work included subjects with other severe diseases involving the heart, liver, brain, and kidney; subjects with mental illness or infectious diseases; woman who is pregnant or breastfeeding; subjects who used other drugs to regulate blood glucose or blood lipids; and subjects who are participating in other clinical experiments or research projects.

### 2.3. Intervention of Hua Tan Qu Shi Recipe

Hua Tan Qu Shi recipe (HTQSR) is a clinical prescription invented by Professor Wang Qi, who is an academician of Chinese Academy of Engineering. The intellectual property right of HTQSR has been protected, and its patent number is ZL201410538335.9. HTQSR is provided by Changchun Lei Yun Shang Pharmaceutical Group Co., Ltd. (Changchun, Jilin, China). All the subjects orally took 10 grams of HTQSR, twice per day. The intervention period of HTQSR was 12 weeks for each individual.

### 2.4. Sample Collection

Before the sample collection, all subjects were instructed to abstain from fatigue and alcohol for five days. For female participants, their samples were collected outside the menstrual periods. Five milliliters of fasting venous blood from all subjects without the treatment of HTQSR was collected in the morning after fasting for eight hours. Similarly, five milliliters of fasting venous blood from all subjects with the treatment of HTQSR for twelve weeks was collected in the morning after fasting for eight hours. Samples were placed for 30 minutes and centrifuged at the speed of 2000 r/min for 10 minutes. Then, the upper plasma was collected and frozen at -80°C.

### 2.5. Sample Preparation

In the 96-well plate (Corning, America), 10 *μ*L of BSA solution or above samples were added. Under the dark conditions, 200 *μ*L of the Bradford solution was added into every well, and the plate was shaken for one minute. The absorbance value at 595 nm was measured with a microplate reader (Genentech, San Francisco, America), and the concentration of every sample was calculated. Then, samples were loaded onto a filter membrane and washed with UA solution (24 g urea and 0.6 g Tris with 50 mL water), 25 mM ammonium bicarbonate solution and 500 mM NaCl solution, respectively. With the speed of 14000 g/min for 10 minutes at 20°C by an ultracentrifuge (Thermo Fisher Scientific, America), peptide solution was prepared. Waters Oasis C18 column was used to extract the above peptide solution. Finally, the concentration of peptide solution was analyzed by the BCA Protein Assay kit (Thermo Pierce, America). The absorbance values of peptide solutions were measured at 562 nm with a microplate reader (Genentech, San Francisco, America).

### 2.6. DIA-MS Proteomic Analysis

#### 2.6.1. Offline High-pH RPLC Separation

0.1% formic acid solution was used to dissolve the above peptide sample, and this system was added into a high-pH reverse phase liquid chromatography (RPLC) column (4.6 mm × 250 mm, XBridge C18, 3 *μ*m; Waters, Milford, America). Buffer A was prepared with deionized water, and its pH was adjusted to 10.0 by ammonia water. Buffer B was prepared with 90.0% acetonitrile solution, and its pH was also adjusted to 10.0 by ammonia water. Peptide samples were eluted using buffer A and buffer B for 60 minutes, and the flow velocity of RPLC separation was 1 mL/min. Finally, the eluted peptide was dissolved into 0.1 formic acid solution and stored at -20°C.

#### 2.6.2. LC-MS/MS Analysis

Orbitrap Fusion Lumos Tribrid mass spectrometer (Thermo Fisher Scientific, Germany) was used to identify peptides. Instrument parameters were set as follows: the first-level full scan range was from 350 m/z to 1550 m/z, and its resolution was 120,000; the second-level scan collision energy was 30%, and its resolution was 30,000; the acquisition method was high-speed mode; the dynamic exclusion time was 30 s; and the maximum ion implantation time was 0.045 s.

Data-independent acquisition (DIA) mode was utilized in the analysis of quality control samples and all experiments. Variable isolation windows were developed for the MS acquisition. Instrument parameters were set as follows: the primary scanning resolution was 120,000; the mass-to-charge ratio ranged from 400 m/z to 900 m/z; the secondary scanning resolution was 30,000; the collision energy was 32%; the AGC target was 1000,000; and the maximum ion implantation time was 0.05 s.

#### 2.6.3. Spectral Library Generation

Mass spectrometric data were processed by the Proteome Discoverer software (Thermo Scientific, America) and searched using the human Swiss-Prot database. The fixed modification was cysteine carbamidomethylation (+58.00 Da). In addition, the variable modifications were the formylation of protein K-terminal (+43.00 Da), the acetylation of protein N-terminal (+0.98 Da), and the oxidation of methionine (+16.00 Da), respectively. Finally, all data were analyzed using Spectronaut Pulsar 12 software (Biognosys, Zurich, Switzerland).

### 2.7. Differentially Expressed Proteins Screening

Spectronaut Pulsar 12 (Biognosys, Zurich, Switzerland) was used to analyze the DIA data, and the retention time of peptide was calculated on the basis of iRT data. Protein samples were quantified and identified by matching the retention time and m/z of peptides. In this work, the set point of correction factors for MS1 and MS2 was 1. The set point of precursor posterior error probability cut-off was also 1. In order to correct the systematic variance from the performance of LC-MS/MS, local normalization and cross-run normalization strategies were utilized. Umetrics SIMCA 14.1 software (Umetrics, Umea Municipality, Sweden) was used to perform the analysis of pattern recognition. MetaboAnalyst online analysis (https://www.metaboanalyst.ca/) was implemented to show the distribution of the differential protein content. Proteins that presented a fold change above 2 and an adjusted *P* value below 0.05 were considered differentially expressed proteins.

### 2.8. Ingenuity Pathway Analysis (IPA) and Venn Analysis

Ingenuity pathway analysis software (Ingenuity Systems, Mountain View, America) was utilized to analyze all differentially expressed proteins. These proteins were ranked by *P* value and *z*-score, respectively. In addition, the ranking proteins will be mapped to function categories, canonical pathways, and diseases. Venn analysis and Venn diagrams were performed according to the reported reference [14].

### 2.9. PPI Network Construction and Hub Protein Screening

Protein-protein interaction (PPI) networks were constructed based on the STRING online database (https://cn.string-db.org/). Then, PPI networks were drawn and analyzed using Cytoscape software (https://cytoscape.org/). The hub proteins in PPI networks were screened and identified using cytoHubba as a plugin in the Cytoscape software.

### 2.10. Parallel Reaction Monitoring (PRM) Analysis

TripleTOF 5600 instrument (AB Sciex, Framingham, America) was used to perform the PRM analysis for the validation of differentially expressed proteins. C18 monolithic capillary column (50 *μ*m × 500 mm) was utilized to separate peptides, and the set point of normalized collision energy was 35%.

Skyline software (Skyline, Boston, America) was used to analyze the MS data. Set points of peptides were listed as follows: maximum missed cleavages were 2; length of peptides was from 8 to 25; trypsin (KR/P) was set for enzymes; and variable modifications were the carbamidomethylation of cysteine and the oxidation of methionine. Transition settings followed these parameters: ion charges of 1 and 2; precursor charges of 2 and 3; and ion types of y and b.

For the data analysis of PRM, correct peaks in the Skyline software (Skyline, Boston, America) were manually selected. Progenesis software (Waters, Milford, America) was used to extract the total ionic chromatography of ions (+2 − +5 charge) from every sample. In order to adjust the signal intensity and the sample loading amount, mass spectra was normalized with the total ionic chromatography strength. Peptides and differential proteins were analyzed and compared with the results of data-independent acquisition. Peptides presented with *P* value below 0.05 and a cutoff value of 1.2-fold change between groups were defined as differentially expressed proteins.

### 2.11. Statistical Analysis

In this work, statistical analysis was processed by the SPSS software (SPSS Inc., Chicago, America). The analysis of blood glucose, blood lipids, age, and BMI was performed by one-way ANOVA or the Kruskal-Wallis test, and the results were exhibited by the standard deviation of mean. Fisher's Exact test was utilized for gender. *t*-test was used to compare the monitored proteins between different groups. A two-sided *P* value of < 0.05 was defined to be statistically significant.

## 3. Results

### 3.1. Clinical Participants

All the 32 clinical participants aged from 18 to 50 years were recruited from communities in Beijing, and approval number of clinical experiments was 2019BZYLL0310. Baseline data of the gender and age were balanced among the W, T, and Z groups without significant differences. In addition, there were significant differences in the blood glucose, blood lipids, and BMI among the W, T, and Z groups. After the clinical participants from phlegm-dampness constitution were intervened by HTQSR for 12 weeks, the glycolipid indexes, including FBG, 2hPBG, and TC, were significantly declined among the W and AW, T and AT, and Z and AZ groups, respectively. HDL-C was increased among all the three groups, and there was no statistical significance. Importantly, the exclusion criteria for participants were also performed. Sample details of clinical participants were exhibited in Table 1.

### 3.2. Differential Plasma Proteins Identified by DIA-MS Proteomics

**Figure 2:**
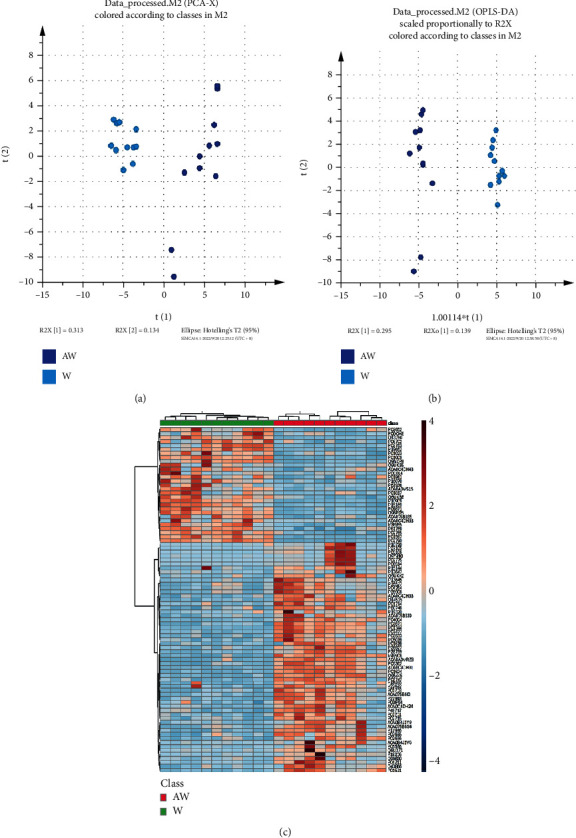
Plasma proteomic analysis of the phlegm-dampness constitution before sickness groups (W/AW) before and after the treatment of the HTQSR. (a) Score plot of the unsupervised PCA model between W and AW; (b) score plot of the supervised OPLS-DA clustering between W and AW; and (c) heat map of the 87 differentially expressed proteins between W and AW. PCA: principal component analysis; OPLS-DA: orthogonal partial least square discriminant analysis.

Results from the score plot of the unsupervised PCA model and the score plot of the supervised OPLS-DA clustering between W and AW in Figures 2(a) and 2(b) demonstrated that there were significant differences between the groups of W and AW. From the results of heat map analysis in Figure 2(c), 87 differentially expressed proteins between W and AW were screened. Among them, 58 differentially expressed proteins were upregulated, and 29 differentially expressed proteins were downregulated.

**Figure 3:**
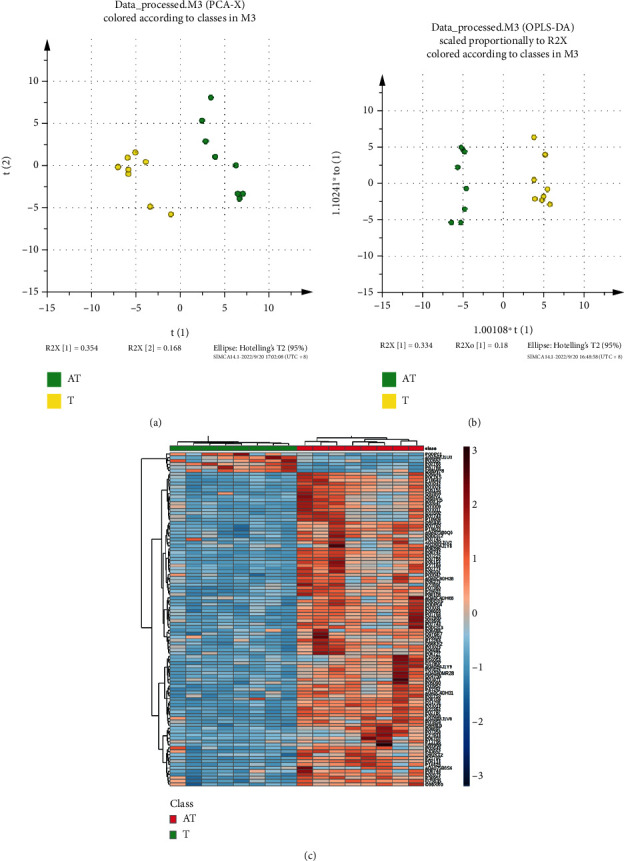
Plasma proteomic analysis of the phlegm-dampness constitution with the prediabetic state groups (T/AT) before and after the treatment of the HTQSR. (a) Score plot of the unsupervised PCA model between T and AT; (b) score plot of the supervised OPLS-DA clustering between T and AT; and (c) heat map of the 102 differentially expressed proteins between T and AT.

Based on the results from the score plot of the unsupervised PCA model and the score plot of the supervised OPLS-DA clustering between T and AT in Figures 3(a) and 3(b), there were significant differences between the groups of T and AT. As shown in the heat map analysis of Figure 3(c), 102 differentially expressed proteins between T and AT were screened. Among them, 96 differentially expressed proteins were upregulated, and 6 differentially expressed proteins were downregulated.

**Figure 4:**
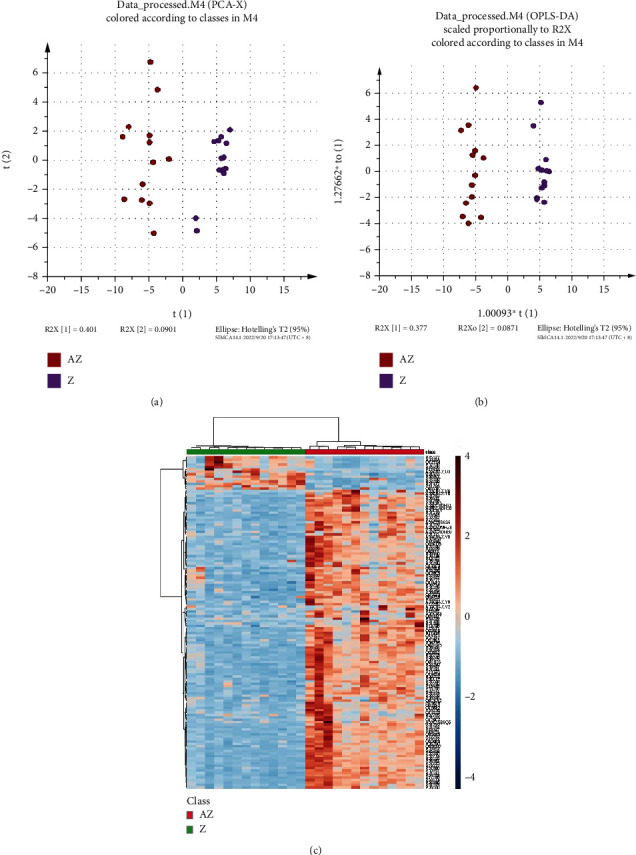
Plasma proteomic analysis of the phlegm-dampness constitution with marginally elevated blood lipids groups (Z/AZ) before and after the treatment of the HTQSR. (a) Score plot of the unsupervised PCA model between Z and AZ; (b) score plot of the supervised OPLS-DA clustering between Z and AZ; and (c) heat map of the 138 differentially expressed proteins between Z and AZ.

According to the results from the score plot of the unsupervised PCA model and the score plot of the supervised OPLS-DA clustering between Z and AZ in Figures 4(a) and 4(b), there were significant differences between the groups of Z and AZ. From the analysis of heat map in Figure 4(c), 138 differentially expressed proteins between Z and AZ were screened. Among them, 124 differentially expressed proteins were upregulated, and 14 differentially expressed proteins were downregulated.

### 3.3. Functional Analysis of Differential Plasma Proteins

**Figure 5:**
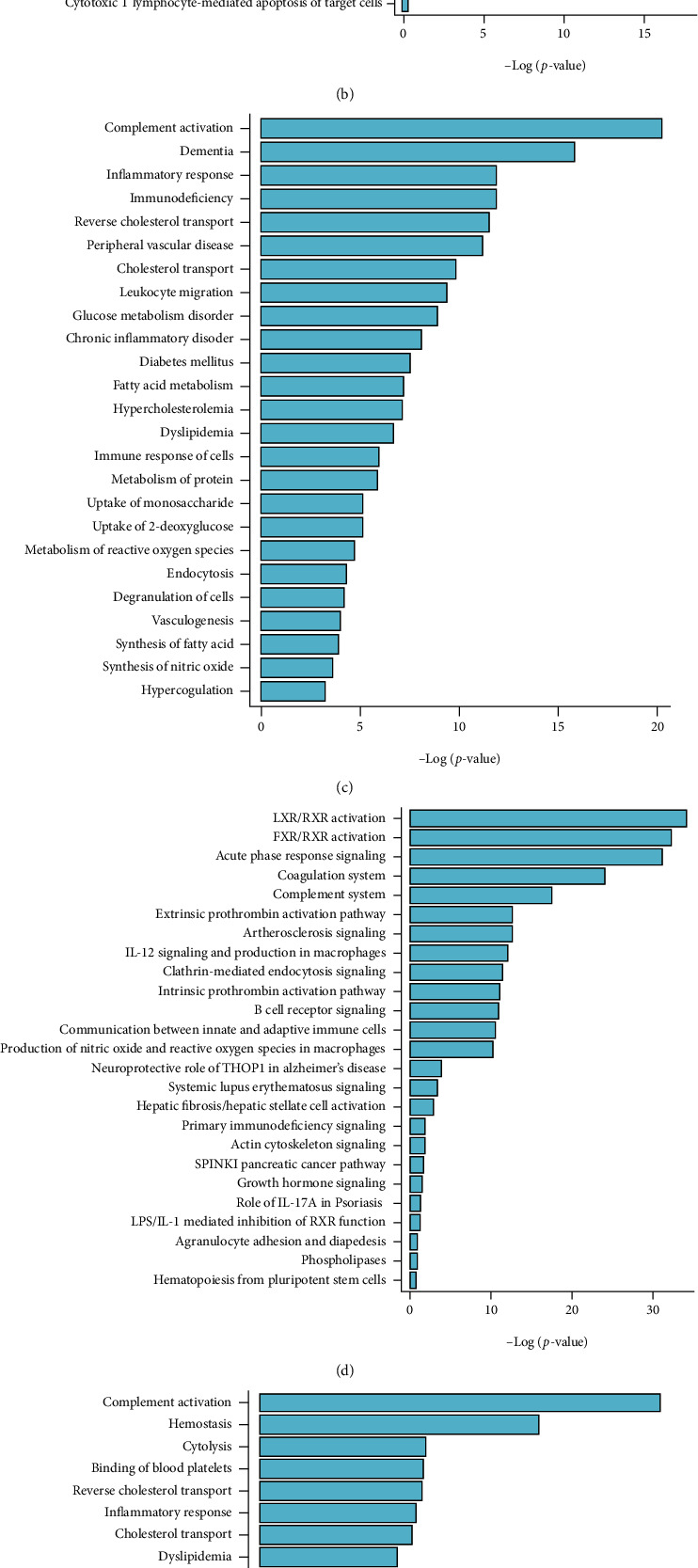
Disease and biofunction analysis and canonical pathway analysis. Disease and biofunction (a) and canonical pathway (b) analysis of the differentially expressed proteins between W and AW; disease and biofunction (c) and canonical pathway (d) analysis of the differentially expressed proteins between T and AT; and disease and biofunction (e) and canonical pathway (f) analysis of the differentially expressed proteins between Z and AZ.

Summary of functional analysis from differential plasma proteins in all groups was displayed in Figure 5. In the disease and biofunction analysis of the differentially expressed proteins between W and AW, proteins associated with complement activation, receptor-mediated endocytosis, transmigration of leukocytes, cell movement, and adhesion of vascular endothelial cells were enriched (Figure 5(a)). The participating pathways between W and AW were LXR/RXR activation, FXR/RXR activation, clathrin-mediated endocytosis signaling, atherosclerosis signaling, and acute phase response signaling, respectively (Figure 5(b)).

In the disease and biofunction analysis of the differentially expressed proteins between T and AT, proteins associated with complement activation, metabolism of protein, leukocyte migration, endocytosis, and inflammatory response were enriched (Figure 5(c)). The participating pathways between T and AT were LXR/RXR activation, FXR/RXR activation, acute phase response signaling, coagulation system, and complement system, respectively (Figure 5(d)).

In the disease and biofunction analysis of the differentially expressed proteins between Z and AZ, proteins associated with complement activation, binding of blood platelets, metabolism of protein, hemostasis, and leukocyte migration were enriched (Figure 5(e)). The participating pathways between Z and AZ were LXR/RXR activation, acute phase response signaling, FXR/RXR activation, coagulation system, and complement system, respectively (Figure 5(f)).

### 3.4. Analysis of Coexpressed Proteins

**Figure 6:**
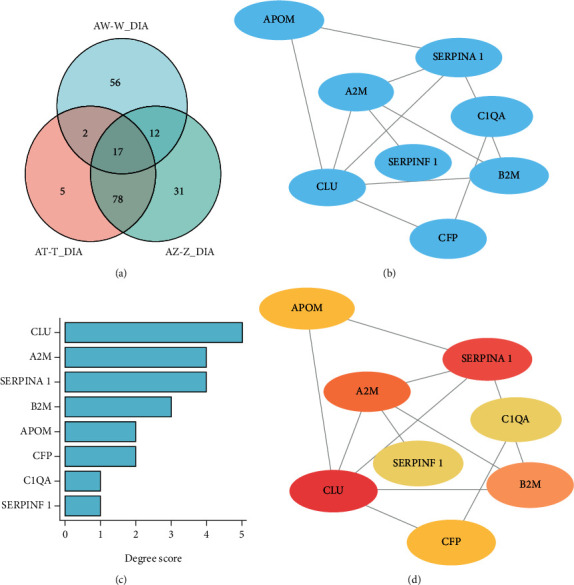
Analysis of coexpressed differential proteins among the AW-W, AT-T, and AZ-Z groups. (a) Venn analysis of coexpressed differential proteins identified by DIA and PPI network analysis (b), degree ranks (c), and hub protein screening (d) of coexpressed differential proteins.

Venn analysis of coexpressed differential proteins identified by DIA indicated that there are 17 common differential proteins among the AW-W, AT-T, and AZ-Z groups in Figure 6(a). Furthermore, PPI network analysis, degree ranks, and hub protein screening of coexpressed differential proteins were performed and showed in Figures 6(b)–6(d), demonstrating that APOM, CLU, SERPINA1, C1QA, B2M, A2M, CFP, and SERPINF1 played an important role in the network.

DIA analysis verified 17 common differential proteins among the AW-W, AT-T, and AZ-Z groups, including KVD30, KVD11, HV118, HV551, APOM, A1AT, A2MG, KV315, HV43D, CLUS, B2MG, PGRP2, HV372, C1QA, PROP, PEDF, and MYH2 (Table 2). Among them, KVD30, KVD11, HV118, HV551, APOM, KV315, HV43D, HV372, C1QA, PROP, and MYH2 were all upregulated in the AW-W, AT-T, and AZ-Z groups. In addition, B2MG was all downregulated in the AW-W, AT-T, and AZ-Z groups. However, A1AT, A2MG, CLUS, PGRP2, and PEDF were upregulated or downregulated in different groups.

### 3.5. Pathway Comparison among Groups

**Figure 7:**
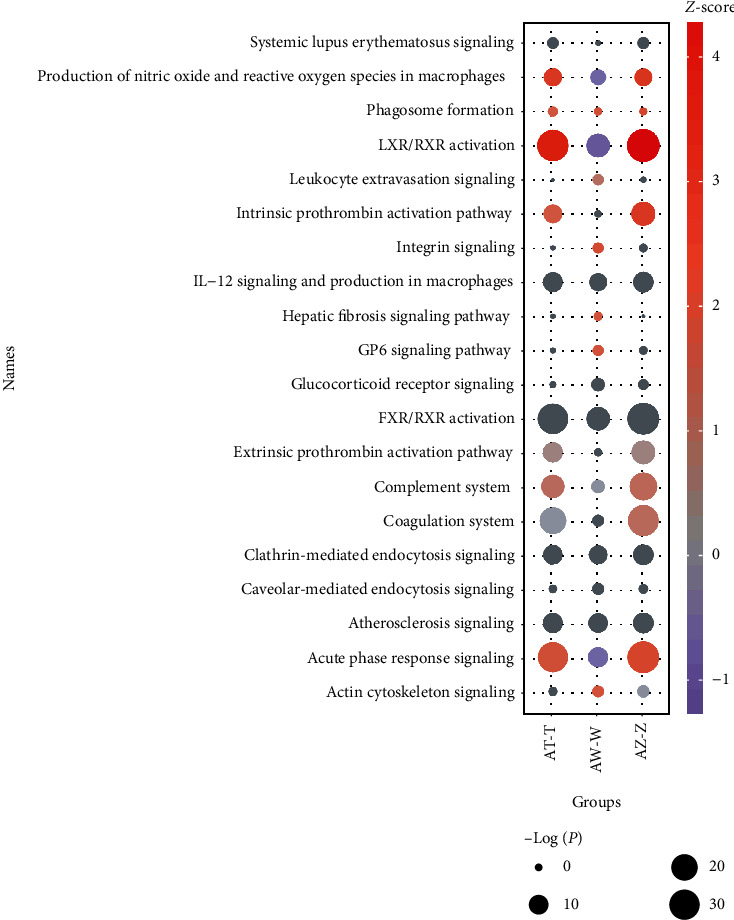
Overlapping pathways among the AW-W, AT-T, and AZ-Z groups by IPA. The color of each circle represents the *z*-score. Red: *z*‐score > 0, activation; blue: *z*‐score < 0, inhibition. The size of each circle represents the −log (*P* value).

Analysis of pathways among the AW-W, AT-T, and AZ-Z groups by IPA is summarized in Table 3 and Figure 7. The *z*-score and −log (*P* value) are exhibited in Figure 7 to indicate the activation or inhibition of pathways. Among them, LXR/RXR activation, acute phase response signaling, and production of nitric oxide and reactive oxygen species in macrophages obviously overlapped in all groups. LXR/RXR activation, acute phase response signaling, production of nitric oxide and reactive oxygen species in macrophages, complement system, and intrinsic prothrombin activation pathway were activated in the AT-T and AZ-Z groups. However, LXR/RXR activation, acute phase response signaling, and production of nitric oxide and reactive oxygen species in macrophages were inhibited in the AW-W group. Integrin signaling and GP6 signaling pathway were activated in the AW-W group.

### 3.6. PRM Validation of Differentially Expressed Proteins

**Figure 8:**
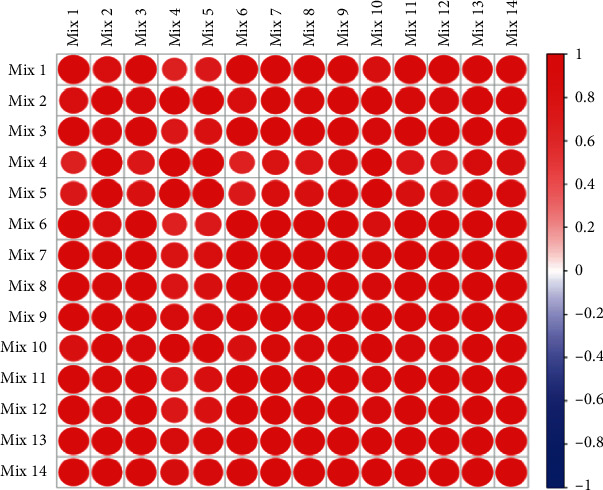
Correlation coefficient map between samples.

A total of 178 differential proteins were quantitatively analyzed. Based on the analysis of the QC samples, the good correlation of QC samples demonstrated that the analysis had a good repeatability, and the MS platform had well stability (Figure 8). In order to validate the differentially expressed proteins, parallel reaction monitoring (PRM) analysis was performed using the TripleTOF 5600 instrument in this work.

According to the analysis results of PRM validation in Table 4, 8 differentially expressed proteins exhibited the same trend as those quantified by the analysis of DIA between the W and AW groups. The value of fold change less than 1.2 in Table 4 represents the downregulated protein expression, and the value of fold change more than 1.2 represents the upregulated protein expression. By the analysis of PRM results from the W and AW groups, there are 2 upregulated differentially expressed proteins and 6 downregulated differentially expressed proteins.

By the analysis results of PRM validation in Table 5, 32 differentially expressed proteins exhibited the same trend as those quantified by the analysis of DIA between the T and AT groups. The value of fold change less than 1.2 in Table 5 represents the downregulated protein expression, and the value of fold change more than 1.2 represents the upregulated protein expression. Based on the PRM results from the T and AT groups, there are 29 upregulated differentially expressed proteins and 3 downregulated differentially expressed proteins.

Based on the analysis results of PRM validation in Table 6, 38 differentially expressed proteins exhibited the same trend as those quantified by the analysis of DIA between the Z and AZ groups. The value of fold change less than 1.2 in Table 6 represents the downregulated protein expression, and the value of fold change more than 1.2 represents the upregulated protein expression. According to the PRM results from the Z and AZ groups, there are 33 upregulated differentially expressed proteins, and 5 downregulated differentially expressed proteins.

To validate the coexpressed differential proteins, PRM analysis was also performed using the TripleTOF 5600 instrument in this work. PRM analysis identified that B2MG was the common differential protein among the AW-W, AT-T, and AZ-Z groups (Table 7). According to the results of PRM analysis, B2MG was all downregulated in the AW-W, AT-T, and AZ-Z groups.

## 4. Discussion

Glucolipid metabolic disorders (GLMD) as a series of states with the metabolic disturbance of lipid and glucose could be affected by genetic, psychological, and environmental factors [15, 16]. Neuroendocrine dysfunction, inflammatory response, insulin resistance, and changes of intestinal flora have been the important pathological phenomena of GLMD [17]. At the same time, diabetes, hypertension, hyperlipidemia, nonalcoholic fatty liver disease, obesity, atherosclerosis, and other clinical manifestations appeared alone or simultaneously [18]. Thus, the treatment of GLMD should be considered comprehensively in clinical practice. Luckily, TCM has great advantages in the preventive and comprehensive treatment of disease before onset.

Constitution in TCM is a relatively integrated and stable intrinsic characteristic of mental state, morphological structure, and physiological function formed by congenital and acquired endowment [19]. According to clinical presentations, TCM constitution could be categorized as nine types: inherited special constitution, balance constitution, blood stasis constitution, damp-heat constitution, yin-deficiency constitution, phlegm-dampness constitution, qi-stagnation constitution, qi-deficiency constitution, and yang-deficiency constitution [20]. Because of the clinical correlation between phlegm-dampness constitution with GLMD, scientific studies of phlegm-dampness constitution have attracted the keen interest of many research groups globally [21]. Based on these findings, we hypothesized that the intervention of phlegm-dampness constitution might provide a novel strategy to treat GLMD.

In this work, HTQSR as a famous formula was used to intervene clinical participants with glucolipid metabolic disorders induced by phlegm-dampness constitution. According to these studies, we found that LXR/RXR activation, acute phase response signaling, and production of nitric oxide and reactive oxygen species in macrophages overlapped among the AW-W, AT-T, and AZ-Z groups. Liver X receptor (LXR) as the nuclear receptor including LXR*α* and LXR*β* plays pivotal roles to regulate cholesterol metabolism and lipid metabolism [22]. Retinoid X receptor (RXR) as the nuclear receptor binding with stereoselectivity the vitamin A derivative 9-*cis* retinoic acid contains RXR*α*, RXR*β*, and RXR*γ* [23]. LXR and RXR could bind with transcription activation regulators to form the heterodimer LXR/RXR for regulating the expression of target genes at transcriptional levels [24]. LXR activation induces cholesterol efflux and exerts anti-inflammatory actions for atherosclerosis with systemic benefits [25]. Regulation of LXR/RXR pathway provides promising therapeutic insight into disorders associated with dysregulated metabolism of lipid and glucose [26]. After the treatment of Hua Tan Qu Shi recipe, LXR/RXR pathway was activated among phlegm-dampness constitution with the prediabetic state and phlegm-dampness constitution with marginally elevated blood lipids. And apolipoprotein-related high-density lipoprotein cholesterol involving in reverse cholesterol transport (RCT) APOA1, APOA4, APOE, APOM, CLU(APOJ), and LCAT were all upregulated (APO3 was obviously downregulated in AZ-Z), which indicated that LXR/RXR activation could promote reverse cholesterol transport.

Acute phase response (APR) is a complex innate immunity system activated by various injuries including stress, trauma, infection, and inflammation [27]. It not only promotes the occurrence and development of inflammation but also can inhibit, reverse, and repair the inflammatory. In essence, APR is a kind of defense and protection effect for the organism. Recent studies reported that C-reactive protein as an acute phase reactant has close relationship with type 2 diabetes [28], as well as the inflammatory marker of cardiovascular diseases. After bariatric surgery, plasma proteomic alternations of remission diabetes proteins participated in acute phase response, platelet degranulation, fibrinolysis, and coagulation in obese patients with T2D [29]. Cardiovascular and metabolic diseases including atherosclerosis and diabetes have a common hallmark endothelial dysfunction, with impaired endothelium-dependent vasodilation, heightened oxidative stress, and chronic inflammation [30]. Macrophage activation produces nitric oxide (NO) and reactive oxygen species (ROS) involved in receptor-mediated phagocytosis, natural defense, and inflammatory response. The appropriate amount of NO can promote endothelial cell-dependent vasodilation, delay the endothelial damage, and lower the blood glucose of DM. Lipids such like cholesterol, fatty acids that modulate inflammatory processes, are signaling molecules for the activation and function of immune cells such as macrophages involved in immune and inflammatory responses [31]. Previous work by Yanagisawa et al. showed that the accumulation of very long chain saturated fatty acids in macrophages enhanced the production of NO, ROS, and proinflammatory cytokines [32]. With the treatment of Hua Tan Qu Shi recipe, acute phase response signaling and production of nitric oxide and reactive oxygen species in macrophages were also activated among phlegm-dampness constitution with the prediabetic state and phlegm-dampness constitution with marginally elevated blood lipids.

With the treatment of HTQSR, disease and function analysis showed that the differential proteins among the W/AW, T/AT, and Z/AZ groups were all enriched in complement activation, leukocyte migration, inflammatory response, immune response of cells, humoral immune response, reverse cholesterol transport, fatty acid metabolism, diabetes mellitus, atherosclerosis, chronic inflammatory disorder, etc. Results in PPI network analysis and hub protein screening of coexpressed differential proteins suggested that APOM, CLU, SERPINA1, C1QA, B2M, A2M, CFP, and SERPINF1 played an important role in the network. These proteins are mostly related to cholesterol metabolism, immune regulation, and inflammation response involved in reverse cholesterol transport, complement activation, and regulation of immune response.

The results above all indicated that phlegm-dampness constitution before sickness and phlegm-dampness constitution with GLMD are involved in immune response, inflammatory response, and glucolipid metabolic disorders. Additionally, it is an interesting phenomenon that LXR/RXR activation, production of nitric oxide and reactive oxygen species in macrophage, and acute phase response signaling pathway were inhibited in W-AW while prominently activated in T-AT and Z-AZ by the intervention of HTQSR. On the one hand, this might be related to the states where they were in. In phlegm-dampness constitution before sickness with metabolically healthy state, other two populations with abnormalities of glucose or (and) lipid metabolism were on the verge of disease incidence. On the other hand, immune response of cells was very slightly inhibited, and inflammatory response was slightly activated in the W-AW, while both immune response of cells and inflammatory response were obviously activated in AT-T and AZ-Z. Another truth, CLU and SERPINA1 of the three pathways were downregulated in W-AW and upregulated in AT-T and AZ-Z, which also could explain the question. Therefore, HTQSR recuperated the different health states of the phlegm-dampness constitution through modulating the activation or inhibition of the common and related pathways of glucose and lipid metabolism, immunity, and inflammation, to keep the body in a balanced state. Furthermore, integrins are involved in cell signal transduction and activation, cell extension, cell movement, differentiation, and inflammation. As cell adhesion receptors, integrins mediate leukocyte migration and functions. Integrin signaling activation has a positive feedback effect on platelet aggregation. In this study, integrin signaling was only activated in the AW-W group by the intervention of HTQSR, which was the unique proteomic feature of phlegm-dampness constitution before sickness.

Based on the DIA-PRM proteomic analysis, there was one obviously coexpressed and coregulated differential proteins among all groups: *beta*-2-microglobulin (B2MG). B2MG, a low-weight molecular serum globulin produced by lymphocytes, platelets, and polymorphonuclear leukocytes, is the light chain of human leukocyte antigen (HLA) class I antigens, which plays an important role in immunomodulation and inflammation. B2MG levels in serum have been effective predictors of all-cause and diabetes-related mortality in patients with diabetes regardless of renal functions [33]. B2MG with higher serum has been reported as an independent risk factor for diabetic nephropathy and subclinical atherosclerosis in T2D patients without renal dysfunction [34]. Clinically healthy obese people might tend to have higher degrees of B2MG than normal-weight people [35]. Moreover, higher B2MG was correlated with a higher occurrence of major adverse cardiac events [36]. And B2MG as a mediator had been linked to the diet and incident cardiovascular disease, also including all-cause mortality [37]. Lately, it was reported that circulating B2MG with higher levels could be a predictor for the progression of diabetic nephropathy in T2D patients [38]. According to the results of PRM analysis, B2MG was all downregulated in the AW-W, AT-T, and AZ-Z groups. These findings indicated that B2MG was obviously regulated by the intervention of phlegm-dampness constitution against glucolipid metabolic disorders. These studies proved multipathway regulation effects of HTQSR and illustrated the possibility and necessity of exploring the precise regulatory mechanisms between the coregulated protein B2MG and the three coregulated pathways.

## 5. Conclusion

With the treatment of Hua Tan Qu Shi recipe, there were alterations of pathways associated with glycolipid metabolism, immune response among phlegm-dampness constitution with glucolipid metabolic disorders. Pathways, including LXR/RXR activation, acute phase response signaling, and production of nitric oxide and reactive oxygen species in macrophages, were obviously overlapped. Compared with the preintervention, B2MG was downregulated in phlegm-dampness constitution before sickness and phlegm-dampness constitution with GLMD by the treatment of HTQSR. In addition, B2MG was related to glycolipid metabolism, immune response, and inflammation response. These results indicated that HTQSR played a regulatory role in GLMD by the regulation of phlegm-dampness constitution with B2MG and pathways at the proteomic level. In conclusion, these proteomic mechanisms discovered valuable insights that the intervention of phlegm-dampness constitution provides a novel strategy for the preventive treatment of GLMD.

## Figures and Tables

**Table 1:** Parameter summary of clinical participants.

Characteristics	W	AW	T	AT	Z	AZ	*P* value
Gender (M/F)	11 (4/7)		8 (4/4)		13 (7/6)		0.679^a^
Age (year)	31.36 ± 9.45		39.88 ± 5.89		33.92 ± 8.29		0.096^b^
BMI (kg/m^2^)	26.80 ± 4.01	26.63 ± 3.90	31.32 ± 3.28	31.39 ± 3.96	28.44 ± 4.13	28.24 ± 4.54	0.033^b^
FBG (m Mol/L)	4.47 ± 0.51	4.18 ± 0.55^c^	5.83 ± 0.65	5.91 ± 1.32	4.42 ± 0.66	4.30 ± 0.29	0.000^b^
2hPBG (m Mol/L)	5.18 ± 0.94	4.66 ± 1.06	9.34 ± 1.14	7.96 ± 2.17^c^	5.10 ± 0.99	4.78 ± 0.91	0.000^b^
TC (m Mol/L)	4.34 ± 0.35	4.52 ± 0.81	5.84 ± 1.35	5.77 ± 0.90	5.25 ± 0.54	4.69 ± 0.90^c^	0.001^b^
TG (m Mol/L)	0.92 ± 0.27	0.96 ± 0.45	4.01 ± 1.24	3.85 ± 1.80	1.43 ± 0.45	1.32 ± 0.54	0.000^b^
HDL-C (m Mol/L)	1.16 ± 0.20	1.27 ± 0.23	1.01 ± 0.20	1.14 ± 0.32	1.23 ± 0.25	1.30 ± 0.30	0.096^b^
LDL-C (m Mol/L)	2.53 ± 0.37	2.68 ± 0.65	3.35 ± 0.97	3.02 ± 0.47	3.17 ± 0.43	2.97 ± 0.59	0.010^b^
Non-HDL-C (m Mol/L)	3.18 ± 0.35	3.25 ± 0.77	4.83 ± 1.28	4.63 ± 0.99	4.02 ± 0.43	3.38 ± 0.92^c^	0.000^b^

BMI: body mass index; FBG: fasting blood glucose; 2hPBG: 2 hours postprandial blood glucose; TC: total cholesterol; TG: triglyceride; HDL-C: high-density lipoprotein cholesterol; LDL-C: low-density lipoprotein cholesterol; ^a^Two-sided *P* value for the Fisher's exact test. ^b^Two-sided *P* value for one-way ANOVA or the Kruskal-Wallis test among the W, T, and Z groups. ^c^Two-sided *P* value for *t*-test between the W and AW, T and AT, and Z and AZ groups, and *P* < 0.05.

**Table 2:** Coexpressed differential proteins identified by DIA analysis among the AW-W, AT-T, and AZ-Z groups.

Number	Protein accession	Protein name	Gene name	Regulated type (AW-W/AT-T/AZ-Z)
1	A0A075B6S6	KVD30_HUMAN	IGKV2D-30	Up
2	A0A0A0MRZ8	KVD11_HUMAN	IGKV3D-11	Up
3	A0A0B4J1Y9	HV372_HUMAN	IGHV3-72	Up
4	A0A0C4DH31	HV118_HUMAN	IGHV1-18	Up
5	A0A0C4DH38	HV551_HUMAN	IGHV5-51	Up
6	O95445	APOM_HUMAN	APOM	Up
7	P01009	A1AT_HUMAN	SERPINA1	Down/up/up
8	P01023	A2MG_HUMAN	A2M	Down/up/up
9	P01624	KV315_HUMAN	IGKV3-15	Up
10	P02745	C1QA_HUMAN	C1QA	Up
11	P0DP04	HV43D_HUMAN	IGHV3-43D	Up
12	P10909	CLUS_HUMAN	CLU	Down/up/up
13	P27918	PROP_HUMAN	CFP	Up
14	P36955	PEDF_HUMAN	SERPINF1	Down/up/up
15	P61769	B2MG_HUMAN	B2M	Down
16	Q9UKX2	MYH2_HUMAN	MYH2	Up
17	Q96PD5	PGRP2_HUMAN	PGLYRP2	Down/up/up

**Table 3:** Overlapping pathways among the AW-W, AT-T, and AZ-Z groups.

Canonical pathways	*z*-score		
	AW-W	AT-T	AZ-Z
LXR/RXR activation	-1.291	3.8	4.271
FXR/RXR activation	N/A	N/A	N/A
Acute phase response signaling	-1	2.111	2.496
Production of nitric oxide and reactive oxygen species in macrophages	-1	2.887	3.051
Clathrin-mediated endocytosis signaling	N/A	N/A	N/A
Coagulation system	N/A	0	1.147
Complement system	0	1.134	1.265
Atherosclerosis signaling	N/A	N/A	N/A
IL-12 signaling and production in macrophages	N/A	N/A	N/A
Intrinsic prothrombin activation pathway	N/A	1.89	2.887
Extrinsic prothrombin activation pathway	N/A	0.447	0.447
Caveolar-mediated endocytosis signaling	N/A	N/A	N/A
Hepatic fibrosis signaling pathway	2	N/A	N/A
Glucocorticoid receptor signaling	N/A	N/A	N/A
GP6 signaling pathway	2	N/A	N/A
Integrin signaling	2.236	N/A	N/A
Leukocyte extravasation signaling	1	N/A	N/A
Neuroinflammation signaling pathway	N/A	N/A	N/A
Actin cytoskeleton signaling	2	N/A	0
Systemic lupus erythematosus signaling	N/A	N/A	N/A
Phagosome formation	2.121	1.89	2.449

Notes: N/A: not applicable; *z*‐score > 0, activation; *z*‐score < 0, inhibition.

**Table 4:** PRM validation of differentially expressed proteins between the W and AW groups.

Number	Protein accession	Protein name	Gene name	Fold change (AW/W)	*P* value
1	P04264	K2C1_HUMAN	KRT1	4.229	2.07*E* − 02
2	P02747	C1QC_HUMAN	C1QC	2.081	2.50*E* − 02
3	P02790	HEMO_HUMAN	HPX	0.495	1.06*E* − 02
4	P19652	A1AG2_HUMAN	ORM2	0.202	1.15*E* − 03
5	P01817	HV205_HUMAN	IGHV2-5	0.151	4.67*E* − 02
6	P61769	B2MG_HUMAN	B2M	0.268	2.83*E* − 02
7	P02652	APOA2_HUMAN	APOA2	0.126	3.37*E* − 04
8	P02763	A1AG1_HUMAN	ORM1	0.027	2.16*E* − 03

**Table 5:** PRM validation of differentially expressed proteins between the T and AT groups.

Number	Protein accession	Protein name	Gene name	Fold change (AT/T)	*P* value
1	P02743	SAMP_HUMAN	APCS	8.218	1.45*E* − 03
2	P22792	CPN2_HUMAN	CPN2	10.589	8.97*E* − 04
3	P06727	APOA4_HUMAN	APOA4	11.350	2.32*E* − 02
4	P05090	APOD_HUMAN	APOD	3.501	2.88*E* − 02
5	P04114	APOB_HUMAN	APOB	3.847	2.49*E* − 03
6	O14791	APOL1_HUMAN	APOL1	5.035	1.13*E* − 03
7	P00742	FA10_HUMAN	F10	2.880	3.17*E* − 02
8	P36955	PEDF_HUMAN	SERPINF1	3.071	4.30*E* − 03
9	P01019	ANGT_HUMAN	AGT	2.936	2.03*E* − 02
10	P18428	LBP_HUMAN	LBP	7.862	2.24*E* − 04
11	P02649	APOE_HUMAN	APOE	3.123	1.48*E* − 02
12	P06396	GELS_HUMAN	GSN	3.451	1.04*E* − 04
13	P43652	AFAM_HUMAN	AFM	4.106	3.26*E* − 05
14	A0A0C4DH68	KV224_HUMAN	IGKV2-24	3.040	7.47*E* − 03
15	P05155	IC1_HUMAN	SERPING1	2.894	1.48*E* − 03
16	P02768	ALBU_HUMAN	ALB	2.561	4.07*E* − 03
17	P02748	CO9_HUMAN	C9	2.423	7.77*E* − 05
18	P01009	A1AT_HUMAN	SERPINA1	2.055	3.29*E* − 02
19	P07358	CO8B_HUMAN	C8B	2.051	3.88*E* − 03
20	P19823	ITIH2_HUMAN	ITIH2	2.001	8.01*E* − 04
21	P07358	CO8B_HUMAN	C8B	2.051	3.88*E* − 03
22	P08603	CFAH_HUMAN	CFH	2.227	7.27*E* − 03
23	P07225	PROS_HUMAN	PROS1	1.922	2.37*E* − 03
24	P0C0L5	CO4B_HUMAN	C4B	2.115	2.18*E* − 02
25	P02787	TRFE_HUMAN	TF	2.143	4.78*E* − 05
26	P01023	A2MG_HUMAN	A2M	1.698	1.57*E* − 02
27	P00748	FA12_HUMAN	F12	1.548	1.01*E* − 02
28	P01011	AACT_HUMAN	SERPINA3	1.531	9.00*E* − 04
29	P10643	CO7_HUMAN	C7	1.501	2.69*E* − 02
30	P02766	TTHY_HUMAN	TTR	0.415	4.65*E* − 02
31	P02652	APOA2_HUMAN	APOA2	0.026	4.96*E* − 02
32	P61769	B2MG_HUMAN	B2M	0.020	7.06*E* − 04

**Table 6:** PRM validation of differentially expressed proteins between the Z and AZ groups.

Number	Protein accession	Protein name	Gene name	Fold change (AZ/Z)	*P* value
1	P22792	CPN2_HUMAN	CPN2	5.486	1.35*E* − 03
2	P02743	SAMP_HUMAN	APCS	4.037	2.63*E* − 02
3	P04114	APOB_HUMAN	APOB	3.919	5.52*E* − 05
4	P07358	CO8B_HUMAN	C8B	4.052	5.12*E* − 05
5	P22352	GPX3_HUMAN	GPX3	2.628	3.75*E* − 02
6	P05543	THBG_HUMAN	SERPINA7	4.241	1.57*E* − 02
7	P05160	F13B_HUMAN	F13B	3.508	2.35*E* − 06
8	P36955	PEDF_HUMAN	SERPINF1	2.413	2.98*E* − 02
9	P43652	AFAM_HUMAN	AFM	2.554	9.26*E* − 03
10	P03951	FA11_HUMAN	F11	3.092	4.40*E* − 04
11	P18428	LBP_HUMAN	LBP	2.657	7.22*E* − 03
12	P02787	TRFE_HUMAN	TF	2.652	9.46*E* − 05
13	P05090	APOD_HUMAN	APOD	2.339	2.31*E* − 02
14	Q14624	ITIH4_HUMAN	ITIH4	2.437	4.26*E* − 02
15	P02768	ALBU_HUMAN	ALB	2.092	2.26*E* − 04
16	P01009	A1AT_HUMAN	SERPINA1	2.511	4.91*E* − 05
17	P06396	GELS_HUMAN	GSN	1.996	2.10*E* − 02
18	P01019	ANGT_HUMAN	AGT	2.472	1.27*E* − 02
19	P07225	PROS_HUMAN	PROS1	2.187	2.28*E* − 03
20	P02671	FIBA_HUMAN	FGA	2.149	1.05*E* − 03
21	P19823	ITIH2_HUMAN	ITIH2	2.321	2.10*E* − 03
22	P05155	IC1_HUMAN	SERPING1	1.916	4.50*E* − 03
23	P27169	PON1_HUMAN	PON1	2.031	1.17*E* − 02
24	P00742	FA10_HUMAN	F10	1.685	2.90*E* − 02
25	P01024	CO3_HUMAN	C3	1.913	9.53*E* − 03
26	P00450	CERU_HUMAN	CP	1.642	4.93*E* − 02
27	P00736	C1R_HUMAN	C1R	1.659	1.78*E* − 03
28	P04003	C4BPA_HUMAN	C4BPA	1.542	6.40*E* − 03
29	P01023	A2MG_HUMAN	A2M	1.460	1.08*E* − 02
30	P01011	AACT_HUMAN	SERPINA3	1.419	1.90*E* − 03
31	P10643	CO7_HUMAN	C7	1.543	1.38*E* − 03
32	P19827	ITIH1_HUMAN	ITIH1	1.499	4.12*E* − 02
33	P00748	FA12_HUMAN	F12	1.280	3.50*E* − 02
34	P04264	K2C1_HUMAN	KRT1	0.192	9.21*E* − 04
35	P02766	TTHY_HUMAN	TTR	0.184	6.11*E* − 04
36	P35908	K22E_HUMAN	KRT2	0.112	3.27*E* − 03
37	A0A0B4J1U3	LV136_HUMAN	IGLV1-36	0.085	7.13*E* − 03
38	P61769	B2MG_HUMAN	B2M	0.019	4.02*E* − 05

**Table 7:** Coexpressed differential protein identified by PRM analysis among the AW-W, AT-T, and AZ-Z groups.

Protein accession	Protein name	DIA fold change			PRM fold change			PRM *P* value		
		AW/W	AT/T	AZ/Z	AW/W	AT/T	AZ/Z	AW/W	AT/T	AZ/Z
P61769	B2MG_HUMAN	0.351	0.222	0.384	0.268	0.020	0.019	2.83*E* − 02	7.06*E* − 04	4.02E-05

## Data Availability

The data used to support the findings of this study are available from the corresponding author upon request.
